# Effects of Levodopa-Carbidopa Intestinal Gel Compared with Optimized Medical Treatment on Nonmotor Symptoms in Advanced Parkinson's Disease: INSIGHTS Study

**DOI:** 10.1155/2022/1216975

**Published:** 2022-11-04

**Authors:** Sun Ju Chung, Matilde Calopa, Maria G. Ceravolo, Nicola Tambasco, Angelo Antonini, K. Ray Chaudhuri, Weining Z. Robieson, Olga Sánchez-Soliño, Cindy Zadikoff, Man Jin, Luigi M. Barbato

**Affiliations:** ^1^Department of Neurology, Asan Medical Center, University of Ulsan College of Medicine, Seoul, Republic of Korea; ^2^Department of Neurology, Hospital Universitari de Bellvitge, L'Hospitalet de Llobregat, Barcelona, Spain; ^3^Department of Experimental and Clinical Medicine, Neurorehabilitation Clinic, University Hospital of Ancona, Università Politecnica Delle Marche, Ancona, Italy; ^4^Movement Disorders Center, Perugia General Hospital and University of Perugia, Perugia, Italy; ^5^Movement Disorders Unit, Department of Neuroscience, University of Padua, Padua, Italy; ^6^Institute of Psychiatry Psychology & Neuroscience, King's College and Parkinson Foundation International Centre of Excellence, King's College Hospital, Denmark Hill, London, UK; ^7^AbbVie Inc., North Chicago, IL, USA

## Abstract

**Background:**

Nonmotor symptoms (NMS) are common in advanced Parkinson's disease (APD) and reduce health-related quality of life.

**Objective:**

The aim of the study was to evaluate levodopa-carbidopa intestinal gel (LCIG) versus optimized medical treatment (OMT) on NMS in APD.

**Methods:**

INSIGHTS was a phase 3b, open-label, randomized, multicenter study in patients with APD (LCIG or OMT, 26 weeks) (NCT02549092). Primary outcomes assessed were total NMS (NMS scale (NMSS) and PD sleep scale (PDSS-2)). Key secondary outcomes included the Unified PD Rating Scale (UPDRS) Part II, Clinical Global Impression of Change (CGI-C), and PD Questionnaire-8 (PDQ-8). Additional secondary measures of Patient Global Impression of Change (PGIC), King's PD Pain Scale (KPPS), and Parkinson Anxiety Scale (PAS) also were evaluated. Finally, safety was assessed.

**Results:**

Out of 89 patients randomized, 87 were included in the analysis (LCIG, *n* = 43; OMT, *n* = 44). There were no significant differences in NMSS or PDSS-2 total score changes (baseline to Week 26) between LCIG and OMT; within-group changes were significant for NMSS (LCIG, *p* < 0.001; OMT, *p* = 0.005) and PDSS-2 (LCIG, *p* < 0.001; OMT, *p* < 0.001). Between-group treatment differences were nominally significant for UPDRS Part II (*p* = 0.006) and CGI-C (*p* < 0.001) at Week 26 in favor of LCIG; however, statistical significance could not be claimed in light of primary efficacy outcomes. PGIC (Week 26) and KPPS (Week 12) scores were nominally significantly reduced with LCIG versus OMT (*p* < 0.001; *p* < 0.05). There were no significant differences in PDQ-8 or PAS. Adverse events (AEs) were mostly mild to moderate; common serious AEs were pneumoperitoneum (*n* = 2) and stoma-site infection (*n* = 2) (LCIG).

**Conclusions:**

There were no significant differences between LCIG versus OMT in NMSS or PDSS-2; both LCIG and OMT groups significantly improved from baseline. AEs were consistent with the known safety profile.

## 1. Introduction

Parkinson's disease (PD) has traditionally been characterized according to the appearance of cardinal motor symptoms; however, nonmotor symptoms (NMS) included in the original PD description by James Parkinson are increasingly recognized as being critical to identifying and treating patients [1, 2]. NMS such as autonomic disorders and sleep disturbances often appear before motor symptoms [3, 4] and can be more detrimental to health-related quality of life [5].

Levodopa is the most efficacious symptomatic treatment regimen in patients with PD and remains the gold standard for treatment [6–9]. However, long-term use of oral levodopa (5 to 10 years) is associated with the development of disabling motor complications, which are often associated with NMS [6, 7]. Furthermore, after oral levodopa administration, unsteady plasma dopamine concentrations are evident due to fluctuating levodopa concentrations, stemming from the short half-life of levodopa combined with variable gastric emptying and intestinal absorption [6, 10–12].

Levodopa-carbidopa intestinal gel (LCIG) is a carboxymethyl-cellulose aqueous gel enteric suspension that is currently approved for use by the US Food and Drug Administration and the EU countries' Mutual Recognition Procedure for the treatment of patients with PD [7, 13–16]. LCIG is continuously delivered to the upper intestine, ensuring plasma concentrations of levodopa remain more stable than with oral administration [7, 12]. A systematic review found that steady plasma levodopa concentrations reduce motor complications and improve control of NMS [17].

At the time of initiation of the current study, few studies had been conducted on both motor and NMS effects of LCIG in patients with PD. One prior randomized study showed significant improvements in motor symptoms and quality of life measures with 12-week LCIG vs. oral levodopa-carbidopa in patients with PD (*n* = 37 and *n* = 34) [18]. In an open-label, phase 3b, 60-week study, patients with APD treated with LCIG showed significant improvement from baseline in NMS scale (NMSS) total scores (*n* = 35 at 12 weeks and *n* = 28 at 60 weeks) [19]. In addition, since the study initiation, a series of observational trials have been completed. Though limited by the open-label, single-arm nature of these studies, they have provided evidence for the reduction of motor complications and improved NMS with LCIG therapy in patients with PD (e.g., GLORIA, DUOGLOBE, MONOTREAT, and COSMOS) [7, 12, 20, 21]. According to these observational studies, 6 to 24 months of LCIG therapy led to improvements in motor fluctuations, including reductions in dyskinesia time and severity, as well as improvements in NMS symptoms, caregiver strain, and other health-related outcomes in patients with advanced PD (APD) [7, 12, 20, 21]. Furthermore, evidence from the retrospective, cross-sectional, postmarketing observational COSMOS study demonstrated that LCIG is a potentially effective long-term monotherapy strategy for the reduction of motor and certain nonmotor symptoms (e.g., anxiety, pain, depression, and constipation) in patients with APD, which may eliminate the need for multiple medications in patients with APD [22].

There continues to be a need for randomized trials comparing LCIG to optimized medical treatment (OMT) in patients with APD. The investigation of NMS in patients with PD is an evolving field; however, clear and widely accepted guidance on how NMS should be evaluated is not yet available. Developments in tools validated for assessing NMS have allowed clinical trials to incorporate nonmotor measures as outcomes [23]. For example, the NMSS, validated in 2007, was developed to capture a broad range of NMS in PD [24]. The PD Sleep Scale-2 (PDSS-2), validated in 2011, serves as a bedside screening tool for identifying sleep problems in PD [25, 26].

The INSIGHTS study aimed to examine the effect of LCIG relative to that of OMT on NMS associated with APD as assessed by NMSS and PDSS-2 total scores. The study also evaluated the impact of LCIG compared with OMT on motor symptoms or complications, health-related outcome measures, safety, and tolerability.

## 2. Materials and Methods

### 2.1. Study Design

**Figure 1:**
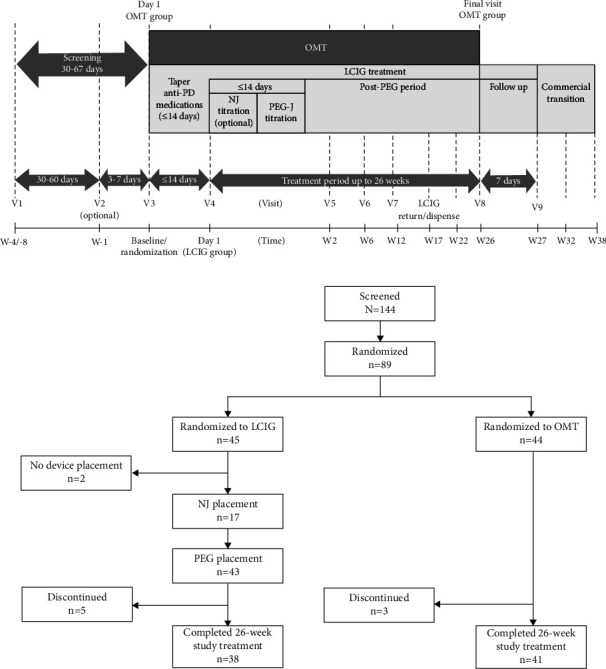
Study design (a) and patient flow diagram (b). Abbreviations: Anti-PD: anti-Parkinson's disease, LCIG: levodopa-carbidopa intestinal gel, NJ: nasojejunal, OMT: optimized medical treatment, PEG-J: percutaneous endoscopic gastronomy with jejunal extension, *V*: visit, and *W*: week.

INSIGHTS was a phase 3b, open-label, randomized, multicenter, 26-week study comparing the effects of LCIG versus OMT on NMS associated with APD (NCT02549092) (Figure 1). All procedures were completed in accordance with the ethical standards of the Independent Ethics Committees/Institutional Review Boards of the institution where data were collected. Patients were randomized in a 1 : 1 ratio, stratified by country, to receive either LCIG or OMT. Patients in the LCIG group must have tapered all anti-PD medications except levodopa formulations (e.g., dopamine agonists, catechol-o-methyl transferase inhibitors, amantadine, monoamine oxidase-B inhibitors, anticholinergics, and subcutaneous apomorphine) within 14 days after randomization so that all medications were stopped before LCIG initiation. All anti-PD medications could be restarted 28 days after LCIG initiation if medically necessary to treat the Parkinsonian symptoms, with the exception of continuous subcutaneous delivery of apomorphine or levodopa-containing formulations.

Patients in the LCIG group received the study drug via percutaneous endoscopic gastronomy with a jejunal extension (PEG-J) tube. An optional temporary nasojejunal (NJ) tube could be used initially with the infusion pump to determine if patients responded favorably to intraintestinal drug delivery and optimize LCIG dose before treatment with a permanent PEG-J tube was started. Once tube placement was confirmed by the investigator, initiation and titration of LCIG infusion started on Day 1. The dose of LCIG was adjusted for an optimal clinical response for each patient over ≤14 days. The optimal clinical response refers to maximizing the functional “On” time during the day by minimizing the number and duration of “Off” episodes (bradykinesia) and minimizing “On” time with disabling dyskinesia. Once optimized, dose adjustments could be made up to Day 28, as needed, at the discretion of the investigator. After Day 28, the LCIG dose was to remain stable for the duration of the study unless adjustments were needed for safety reasons, as discussed with the clinical trial medical monitor. The total daily dose was composed of three components: morning dose, continuous maintenance infusion dose (approximately 16 consecutive waking hours each day starting with a morning dose), and extra doses. Patients had visits at Weeks 2, 6, 12, 17, 22, 26, and 27 (follow-up) (Figure 1(a)).

Patients in the OMT group remained on their current optimized treatment regimen and did not receive NJ/PEG-J tube placement. Study visits occurred at the end of Weeks 2, 6, 12, and 26 following randomization (Figure 1(a)). During the treatment phase, changes to OMT, including adjustments to anti-PD and NMS medications, were to be made if medically justified or if indicated for serious safety reasons.

In calculating sample size, parameters were set as follows: NMSS total score improvement of (mean ± SD) 8.2 ± 24 in OMT and 24.8 ± 24 in LCIG, PDSS-2 total score improvement of (mean ± SD) 4.3 ± 12.2 in OMT and 13.1 ± 12.2 in LCIG. From these criteria, it was determined that 44 patients per group would provide 90% power to declare statistical significance on at least one of these two alternative primary endpoints, after multiplicity adjustment using the Hochberg procedure with the further assumption of a 10% dropout rate.

### 2.2. Participants

Patients with APD (aged ≥30 years) were included. Inclusion criteria included the following: diagnosis of advanced levodopa-responsive PD with persistent motor fluctuations despite optimized therapy and no further improvement expected by the investigator regardless of any additional manipulations of levodopa and/or other antiparkinsonian medication; minimum PDSS-2 total score of 18 at baseline; able to complete the dosing diary; and able to provide informed consent as approved by the Independent Ethics Committees/Institutional Review Board or have informed consent signed by a legal representative. Patients were excluded for the following reasons: PD diagnosis was unclear; suspicion that the patient had parkinsonian syndrome; previous surgery for PD; current or history of significant sleep attacks, impulsive behavior, psychosis, or delusions within three months before screening; had any neurological deficit that might interfere with study assessments, and had vitamin deficiencies (e.g., low B-12 level or low/normal B-12 level (<300 pg/mL) with elevated methylmalonic acid). In addition, neurologic examinations provided continuous monitoring for the onset or worsening of potential peripheral neuropathy throughout the study due to the high prevalence in patients with PD [27]. Though patients were monitored for peripheral neuropathy, they were not excluded from the study due to the benefit: risk relationship and monitoring/treatment techniques at the time of the study.

### 2.3. Assessments

#### 2.3.1. Efficacy

Efficacy variables were measured in Weeks 6, 12, and 26. Baseline values for efficacy variables were evaluated at randomization prior to anti-PD medication tapering and LCIG initiation. The two alternative primary endpoints were the change from baseline to Week 26 in NMSS total score and PDSS-2 total score. The key secondary endpoints were related to motor and health-related outcomes and included the following: change from baseline to Week 26 in Unified PD Rating Scale (UPDRS) Part II and PD Questionnaire-8 item summary index (PDQ-8), and Clinical Global Impression of Change (CGI-C) at Week 26. Additional secondary endpoints associated with health-related outcomes included: Patient Global Impression of Change (PGIC) at Week 26; and change from baseline to Week 26 in King's PD Pain Scale (KPPS) and Parkinson Anxiety Scale (PAS). NMSS, PDSS-2, UPDRS Part II, CGI-C, PDQ-8, PGIC, KPPS, and PAS scales were performed by approved, trained raters with current valid Rater Certificates. For NMSS and PDSS-2, central raters were trained and blinded specialists on movement disorders with previous experience using the NMSS and PDSS-2 scales in patients with APD. For efficacy assessments, the treatment period began the day after randomization (V3) for patients randomized to OMT and the day of the first LCIG infusion following PEG-J placement for patients randomized to LCIG treatment.

#### 2.3.2. Safety

All safety assessments were measured and recorded at baseline and in Weeks 2, 6, 12, and 26, unless otherwise noted. All adverse events (AEs) were monitored and coded using the Medical Dictionary for Regulatory Activities (MedDRA) version 23.0. Sleep attacks were assessed via the Sleep Attacks Questionnaire (SAQ). Additional safety assessments included the Minnesota Impulsive Disorders Interview (MIDI) and Columbia-Suicide Severity Rating Scale (C-SSRS). Clinical laboratory evaluations were measured and recorded at baseline and Weeks 12 and 26 included special laboratory parameters to detect vitamin deficiencies: vitamin B6, vitamin B12, serum folate, methylmalonic acid, and homocysteine. Changes in vital signs (heart rate and blood pressure) were evaluated.

### 2.4. Statistical Analysis

The study was designed to enroll approximately 88 patients to provide sufficient power to declare statistical significance on at least one of the two alternative primary endpoints after multiplicity adjustment. Primary efficacy analyses utilized a likelihood-based mixed-effects model repeated-measures (MMRMs) analysis of the change from baseline for each postbaseline observation using all observed data. An analysis of variance containing treatment and country as the main effects was performed for CGI-C and PGIC. Study results were determined to be statistically significant if at least one of the two primary endpoints was significant at the two-sided 0.05 level after multiplicity adjustment by the Hochberg procedure.

## 3. Results

### 3.1. Patients

The study was conducted at 32 sites in nine countries. Out of 144 patients screened, 89 were randomized and 87 were included in the analysis (LCIG, *n* = 43; OMT, *n* = 44) (Figure 1(b)). Two patients randomized to LCIG did not have devices placed; thus, they did not receive study treatment and were not included in the analysis. Patients were mostly male (LCIG, 67.4% and OMT, 54.5%), with a mean (standard deviation (SD)) age of 66.9 (7.3) years for the LCIG group and 68.6 (6.2) years for the OMT group (Table 1). The mean (SD) years of PD duration since diagnosis was 11.7 (4.9) for the LCIG group and 11.9 (6.0) for the OMT group (Table 1). Patients had mean (SD) baseline NMSS total scores of 99.7 (46.5) (LCIG group) and 112.4 (51.4) (OMT group) (Table 2). Mean (SD) PDSS-2 scores at baseline were 29.9 (7.4) and 30.3 (8.6) for LCIG and OMT groups, respectively (Table 2).

### 3.2. Efficacy

#### 3.2.1. Primary Endpoints: NMS

**Figure 2:**
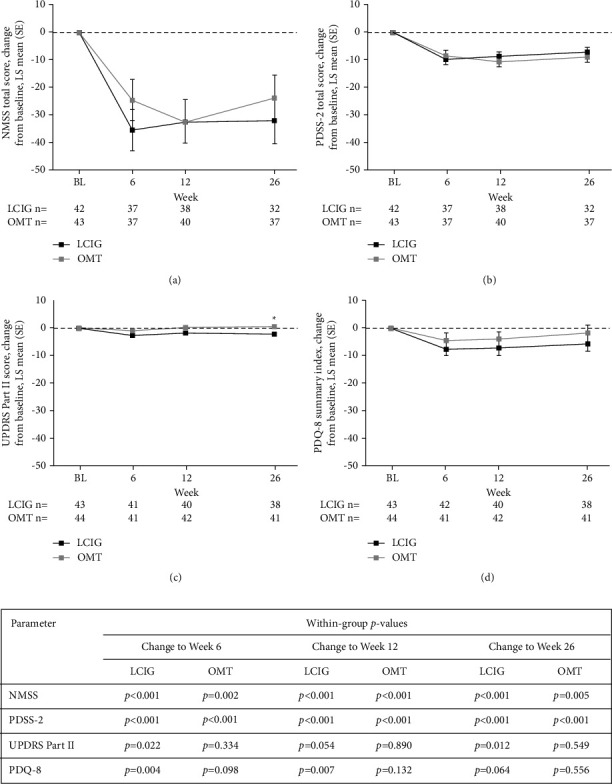
Change from baseline to Week 26 in NMSS (a), PDSS-2 (b), UPDRS Part II (c), and PDQ-8 (d) in the intent-to-treat population. ^*∗*^Nominal between-group difference, *p* < 0.01. Within-group differences versus baseline are noted in table, with significant *p*-values at *p* < 0.05 in bold. LCIG: levodopa-carbidopa intestinal gel, LS: least squares, NMSS: Non-Motor Symptom Scale, OMT: optimized medical treatment, PDSS-2: Parkinson's Disease Sleep Scale, PDQ-8: Parkinson's Disease Questionnaire-8 item summary index, SE: standard error, and UPDRS: Unified Parkinson's Disease Rating Scale.

The two alternative primary endpoints included the change from baseline to Week 26 in NMS measures of NMSS total score and PDSS-2 total score. Between-group comparisons of LCIG versus OMT revealed no significant differences in the changes from baseline to Week 26 in NMSS or PDSS-2 (Table 2; Figures 2(a) and 2(b)). Both treatment groups demonstrated significant reductions from baseline in NMS throughout the study. Mean (SD) NMSS scores reduced significantly in the LCIG group (–30.7 (52.8), *p* < 0.001) and the OMT group (–32.9 (64.3), *p*=0.005); and mean (SD) PDSS-2 scores also reduced significantly in the LCIG group (–7.9 (11.4), *p* < 0.001) and the OMT group (–9.8 (12.8), *p* < 0.001) (Table 2; Figures 2(a) and 2(b)).

#### 3.2.2. Secondary Endpoints: Motor/Health-Related Outcomes

**Figure 3:**
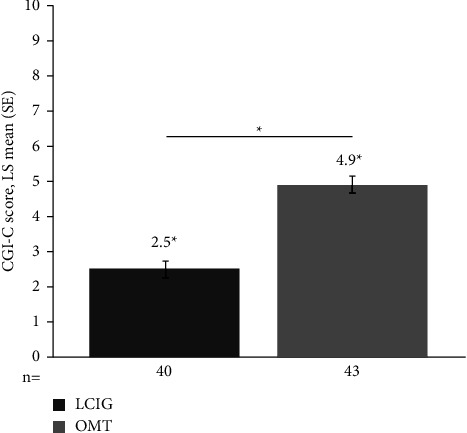
Change at Week 26 in CGI-C in the intent-to-treat population. ^*∗*^Significance for between-group differences (nominal) and within-group differences at Week 26, *p* < 0.001. CGI-C: Clinical Global Impression of Change, LCIG: levodopa-carbidopa intestinal gel, LS: least squares, OMT: optimized medical treatment, and SE: standard error.

The key secondary endpoints included the UPDRS Part II, PDQ-8, and CGI-C. Nominal significance was demonstrated for the change from baseline to Week 26 in UPDRS Part II score, which favored treatment with LCIG versus OMT (least squares (LS) mean (standard error; SE) = –2.3 (0.9) vs. 0.5 (0.9), *p*=0.006; Table 2, Figure 2(c)); however, statistical significance could not be claimed in light of primary efficacy outcomes. No significant differences were observed in the PDQ-8 summary index during the 26-week treatment period (Table 2, Figure 2(d)). The final CGI-C score demonstrated nominal significance, favoring LCIG versus OMT (LS mean (SE) = 2.5 (0.2) vs. 4.9 (0.3), *p* < 0.001; Table 2, Figure 3); again, statistical significance could not be claimed in light of primary efficacy outcomes.

Further secondary endpoints included additional UPDRS scores, PGIC, KPPS, and PAS scores. Between-group comparisons of LCIG versus OMT found no significant differences in the changes from baseline to Week 26 in total UPDRS score, Part I or Part III. However, the nominally significant reduction from baseline to Week 26 in UPDRS Part IV was larger for LCIG versus OMT (LS mean (SE) = −2.3 (0.5) vs. −0.6 (0.5), *p* = 0.007. PGIC score was significantly reduced at Week 26 with LCIG as compared to OMT (LS mean (SE) = 2.5 (0.2) vs. 4.9 (0.3), *p* < 0.001) (Supplemental Figure 1). KPPS showed a nominally significant reduction from baseline to Week 12 with LCIG versus OMT (LS mean (SE) = –15.6 (2.7) vs. –8.0 (2.8), *p* = 0.022), with no significant differences at other time points (Supplemental Figure 2A). PAS showed no significant differences in changes from baseline between LCIG and OMT groups at any time point throughout the study (Supplemental Figure 2B).

#### 3.2.3. Safety

Overall, 36 patients (83.7%) in the LCIG group and 21 patients (47.7%) in the OMT group reported AEs. The most commonly reported treatment-emergent AEs (occurring in >10% of patients) were fall (LCIG, 11.6% and OMT, 15.9%), stoma-site infection (LCIG, 16.3% and OMT, 0%), stoma-site pain (LCIG, 14.0% and OMT, 0%), and depression (LCIG, 11.6% and OMT, 0%). Overall, eight patients (18.6%) in the LCIG group and four patients (9.1%) in the OMT group reported a serious AE (SAE) during the 26-week treatment period. The most frequent SAEs were pneumoperitoneum and stoma-site infection, each of which was reported by two patients (4.7%) in the LCIG group (Table 3); SAEs in the OMT group were bacteremia, urinary tract infection, femur fracture, and PD (all 2.3%).

Overall, seven patients (16.3%) in the LCIG group and eight patients (18.2%) in the OMT group reported one or more sleep attacks during the 26-week treatment period (Supplemental Table 1). There were no positive screens reported for any MIDI module during the 26-week treatment in the LCIG group, and four patients (9.1%) in the OMT group had a positive screen during the 26-week treatment (Supplemental Table 2). According to affirmative responses for the C-SSRS, the number of patients with suicidal behaviors or ideations was similar between the LCIG and OMT groups during the 26-week treatment (Supplemental Table 3).

There were numerical reductions in vitamin B6 with LCIG versus OMT at Week 12 (LS mean (SE) of difference = –12.6 (17.8)) and Week 26 (LS mean (SE) of difference = –31.8 (17.0)) (Supplemental Table 4); there was a significant reduction in the maximum vitamin B6 concentration after LCIG versus OMT (LS mean (SE) of difference = –32.2 (16.0), *p*=0.048). Changes in homocysteine from baseline to Weeks 12 and 26 were significantly different for LCIG versus OMT, with increased mean homocysteine in the LCIG group and reduced mean homocysteine in the OMT group (Week 12, LS mean (SE) of difference = 4.3 [1.5], *p*=0.004, and Week 26, LS mean (SE) of difference = 5.1 (2.0), *p*=0.012) (Supplemental Table 4). There were no statistically significant differences between LCIG and OMT in mean changes in vitamin B12, serum folate, or methylmalonic acid (Supplemental Table 4).

There were no significant changes from baseline in orthostatic hypotension-related vital signs at Weeks 12 or 26 (Supplemental Table 4). Discontinuations during the 26-week treatment period were primarily due to AEs (LCIG, 4.7% and OMT, 4.5%), withdrawal of consent (LCIG, 2.3% and OMT, 2.3%), or a lack of efficacy (LCIG, 4.7% and OMT, 0%).

## 4. Discussion

Within-group differences demonstrated significant improvements in NMSS and PDSS-2 with LCIG and OMT; however, a significant change from baseline to Week 26 with LCIG vs. OMT was not met with either alternative primary endpoint in NMSS or PDSS-2. There was a greater improvement with LCIG versus OMT for key secondary endpoints of UPDRS Part II (change from baseline to Week 26) and CGI-C (Week 26) scores. Additional secondary endpoints of PGIC (Week 26) and KPPS (change from baseline to Week 12) scores demonstrated significant reductions with LCIG compared with OMT. There were no differences in AEs as compared to the known safety profile of LCIG. The SAEs with LCIG were typical of device-related procedures (e.g., pneumoperitoneum and stoma-site infection), while the SAEs with OMT were bacteremia, urinary tract infection, femur fracture, and PD. INSIGHTS demonstrated that LCIG significantly improved NMS (NMSS, PDSS-2) and motor/health-related outcomes (UPDRS Part II, CGI-C, PGIC, and KPPS) from baseline scores at various time points, and was generally safe and well-tolerated.

Results on the improvement of NMS and motor symptoms from baseline with LCIG from INSIGHTS are in line with findings of other studies (e.g., GLORIA [7], DUOGLOBE [20], and MONOTREAT [12]) in which six to 24 months of treatment with LCIG improved motor complications and NMS in patients with PD, as demonstrated by significant changes from baseline in NMSS, PDQ-8, and UPDRS Part II [7, 12, 20]. The randomized design and wide range of endpoints used in INSIGHTS lend further validity to these findings obtained from studies with observational designs.

There are several limitations of the current study, which may have impacted its outcome. One of the study's main limitations is the nonsystematic capture of add-on medications, both dopaminergic and nondopaminergic, which limits the interpretation of their influence on current findings. The study was initially set up to allow anti-PD medications (except for subcutaneous apomorphine or levodopa-containing formulations) to be restarted after Day 28; thus, the focus on capturing data on add-on medications was on anti-PD medications and not the capture of all medications. In addition, the patient population included in the study was somewhat heterogeneous based on the wide range of NMSS scores. Though PDSS-2 was part of the inclusion criteria for INSIGHTS, patient selection for future studies would benefit from the enrollment of patients enriched for selected NMS who are likely to improve with levodopa optimization. Other limitations may stem from the impact of frequent monitoring and the placebo effect associated with the potential benefits and expectations of clinical trial participation in the OMT group. Additionally, OMT may not have been the most optimal comparator because of the current lack of evidence for NMS benefits with OMT. Furthermore, the study ultimately was not powered as planned since treatment differences were lower and the observed variance in NMSS scores was higher than those assumed to estimate sample size requirements. The open-label design and potential interrater variability in the application of study scales are variables that may have impacted study results. Therefore, the effect on outcomes of other medications on NMS could not be assessed. Future randomized trials are needed that focus on patients enriched for NMS that is clearly defined by dopaminergic or nondopaminergic responses; incorporate the use of updated, fully validated tools for the measurement of NMS (e.g., updated questionnaire); and evaluate the impact of the change in medications and doses throughout treatment. Furthermore, LCIG was delivered only during the waking day, thus greater sleep effects may have been reported if administered during nighttime. As an inherent aspect of the treatment, the LCIG group had the NJ or PEG-J placement, a procedure requiring surgery, which may have introduced AEs or side effects that may have impacted sleep and quality of life or PD-related symptoms or introduced bias due to the unblinded design. A follow-up duration of over 26 weeks with a blinded design in terms of NJ or PEG-J placement may be beneficial in future studies in determining differences between LCIG and OMT groups.

## 5. Conclusions

The INSIGHTS study demonstrated comparable improvement of NMS with LCIG therapy versus OMT in patients with APD over the course of 26 weeks. LCIG is a potential alternative treatment strategy to OMT for the control of long-term NMS and motor symptoms in patients with APD and may also offer benefits to health-related quality of life. Though NMS was included in the original description of PD by James Parkinson [1, 2], the study of NMS in patients with PD is not yet well-established. Further support for NMS assessment approaches is needed to guide future investigations. Additional studies with longer follow-up periods are required to understand the differences in NMS control with LCIG versus oral levodopa in both an APD patient population enriched for NMS and in an APD heterogeneous population.

## Figures and Tables

**Table 1:** Baseline demographics and disease characteristics in the safety population.

Demographic and baseline characteristics	LCIG *n* = 43	OMT *n* = 44
Male, *n* (%)	29 (67.4)	24 (54.5)
*Race, n (%)*		
White	34 (79.1)	35 (79.5)
Black	1 (2.3)	0
Asian	8 (18.6)	9 (20.5)
Other	0	0
Age (years), mean (SD)	66.9 (7.3)	68.6 (6.2)
Age ≥65 years, *n* (%)	30 (69.8)	31 (70.5)
Age ≥75 years, *n* (%)	7 (16.3)	7 (15.9)
UPDRS total score (parts I, II, III), mean (SD)	45.5 (18.0)	47.5 (14.9)
UPDRS IV score, mean (SD)	9.1 (3.4)	8.5 (3.2)
PD duration since diagnosis (years), mean (SD)	11.7 (4.9)	11.9 (6.0)
Time since motor fluctuation onset (years), mean (SD)	5.9 (3.6)	6.1 (4.9)
MMSE, mean (SD)	27.5 (1.74)	27.7 (1.76)

BMI: body mass index, LCIG: levodopa-carbidopa intestinal gel, MMSE: Mini-Mental State Examination, OMT: optimized medical treatment, PD: Parkinson's disease, SD: standard deviation, and UPDRS: Unified Parkinson's Disease Rating Scale.

**Table 2:** Primary and key secondary endpoints (intent-to-treat population).

Endpoints^†^		*Observed mean (SD)*						*Between-group comparisons (LCIG vs. OMT)*		
		Group	*n * ^‡^	Baseline	Change to week 26	LS mean (SE)	Within-group *p* value	LS mean (SE) of difference	95% CI	*p* value
*Primary*	NMSS total score	LCIG	42	99.7 (46.5)	−30.7 (52.8)	−32.0 (8.5)	<0.001	−8.2 (9.9)	(−28.0, 11.6)	0.410
		OMT	43	112.4 (51.4)	−32.9 (64.3)	−23.8 (8.3)	0.005			
	PDSS-2 total score	LCIG	42	29.9 (7.4)	−7.9 (11.4)	−7.4 (2.0)	<0.001	1.6 (2.4)	(−3.2, 6.3)	0.509
		OMT	43	30.3 (8.6)	−9.8 (12.8)	−9.0 (2.0)	<0.001			

*Key secondary*	UPDRS part II (ADL) score	LCIG	43	16.7 (7.1)	−2.6 (5.7)	−2.3 (0.9)	0.012	−2.8 (1.0)	(−4.8, −0.8)	0.006
		OMT	44	17.5 (7.0)	−0.1 (4.7)	0.5 (0.9)	0.549			
	PDQ-8 summary index	LCIG	43	39.7 (17.3)	−7.2 (18.3)	−5.6 (3.0)	0.064	−3.8 (3.6)	(−11.0, 3.3)	0.291
		OMT	44	44.5 (17.3)	−6.3 (19.6)	−1.8 (3.0)	0.556			
	CGI-C score	LCIG	40		2.4 (1.2)	2.5 (0.2)	<0.001	−2.3 (0.3)	(−2.8, −1.8)	<0.001
		OMT	43		4.7 (1.2)	4.9 (0.3)	<0.001			

^†^Analyses for all continuous efficacy endpoints are for the change from baseline value (with the exception of CGI-C, which is the score at Week 26). Results for all efficacy variables are based on MMRM analysis with the exception of CGI-C which is based on the ANOVA model. ^‡^Number of patients in the intent-to-treat dataset with the baseline value. Abbreviations: ADL: activities of daily living, ANOVA: analysis of variance, CGI-C: Clinical Global Impression of Change, CI: confidence interval, LCIG: levodopa-carbidopa intestinal gel, LS: least squares, MMRM: mixed-effects model repeated-measures, NMSS: Non-Motor Symptom Scale, OMT: optimized medical treatment, PDSS-2: Parkinson's Disease Sleep Scale, PDQ-8: Parkinson's Disease Questionnaire-8 item summary index, SD: standard deviation, SE: standard error, and UPDRS: Unified Parkinson's Disease Rating Scale.

**Table 3:** Summary of serious AEs in the safety population.

	LCIG *n* = 43	OMT *n* = 44
Any AE	36 (83.7)	21 (47.7)
Any serious AE	8 (18.6)	4 (9.1)
*Treatment-emergent serious AEs*		
Pneumoperitoneum	2 (4.7)	0
Stoma site infection	2 (4.7)	0
Bacteremia	0	1 (2.3)
Urinary tract infection	0	1 (2.3)
Femur fracture	1 (2.3)	1 (2.3)
Parkinson's disease	0	1 (2.3)
Fall	1 (2.3)	0
Lower limb fracture	1 (2.3)	0
Patella fracture	1 (2.3)	0
Subdural hematoma	1 (2.3)	0
Neuralgia	1 (2.3)	0
Depression	1 (2.3)	0

Data are reported as *n* (%). Patients are counted once in each row, regardless of the number of events they may have had. Abbreviations: AE: adverse event, LCIG: levodopa-carbidopa intestinal gel, and OMT: optimized medical treatment.

## Data Availability

AbbVie is committed to responsible data sharing regarding the clinical trials we sponsor. This includes access to anonymized, individual, and trial-level data (analysis data sets), as well as other information (e.g., protocols and clinical study reports), as long as the trials are not part of an ongoing or planned regulatory submission. This includes requests for clinical trial data for unlicensed products and indications. This clinical trial data can be requested by any qualified researchers who engage in rigorous, independent scientific research, and will be provided following review and approval of a research proposal and Statistical Analysis Plan (SAP) and execution of a Data Sharing Agreement (DSA). Data requests can be submitted at any time and the data will be accessible for 12 months, with possible extensions considered. For more information on the process, or to submit a request, visit the following link: https://www.abbvie.com/our-science/clinical-trials/clinical-trials-data-and-information-sharing/data-and-information-sharing-with-qualified-researchers.html.

## Supplementary Materials

Supplementary Materials Supplemental Table 1: Sleep attacks reported at baseline and during the 26-week treatment period in the safety population. Supplemental Table 2: Positive screens reported for each MIDI module at baseline and during the 26-week treatment period in the safety population. Supplemental Table 3: Affirmative responses reported for C-SSRS at baseline and during the 26-week treatment period in the safety population. Supplemental Table 4: Change from baseline to Weeks 12 and 26 in clinical laboratory evaluations and vital signs in the safety population. Supplemental Figure 1: Change at Week 26 in PGIC in the intent-to-treat population. Supplemental Figure 2: Change from baseline to Week 26 in KPPS (A) and PAS (B) in the intent-to-treat population.
