# Supplementary material for: Effects of Levodopa-Carbidopa Intestinal Gel Compared with Optimized Medical Treatment on Nonmotor Symptoms in Advanced Parkinson's Disease: INSIGHTS Study
Source: Parkinsons Dis. 2022 Nov 4;2022:1216975. doi: 10.1155/2022/1216975 (PMC9652073; doi:10.1155/2022/1216975)
Supplement: Supplementary Materials — Supplemental Table 1: Sleep attacks reported at baseline and during the 26-week treatment period in the safety population. Supplemental Table 2: Positive screens reported for each MIDI module at baseline and during the 26-week treatment period in the safety population. Supplemental Table 3: Affirmative responses reported for C-SSRS at baseline and during the 26-week treatment period in the safety population. Supplemental Table 4: Change from baseline to Weeks 12 and 26 in clinical laboratory evaluations and vital signs in the safety population. Supplemental Figure 1: Change at Week 26 in PGIC in the intent-to-treat population. Supplemental Figure 2: Change from baseline to Week 26 in KPPS (A) and PAS (B) in the intent-to-treat population. [file 1216975.f1.docx]

# Supplementary Materials

**Supplemental Table 1.** Sleep attacks reported at baseline and during the 26-week treatment period in the safety population

**Supplemental Table 2.** Positive screens reported for each MIDI module at baseline and during the 26-week treatment period in the safety population

**Supplemental Table 3.** Affirmative responses reported for C-SSRS at baseline and during the 26-week treatment period in the safety population

**Supplemental Table 4.** Change from baseline to Weeks 12 and 26 in clinical laboratory evaluations and vital signs in the safety population

**Supplemental Figure 1.** Change at Week 26 in PGIC in the intent-to-treat population

**Supplemental Figure 2.** Change from baseline to Week 26 in KPPS (A) and PAS (B) in the intent-to-treat population

**Supplemental Table 1.** Sleep attacks reported at baseline and during the 26-week treatment period in the safety population

| **Sleep attacks, n (%)** | **Baseline** | | **During 26-week treatment** | |
| --- | --- | --- | --- | --- |
|  | **LCIG**  **n=43** | **OMT**  **n=44** | **LCIG**  **n=43** | **OMT**  **n=44** |
| 1 or more sleep attacks | 8 (18.6) | 13 (29.5) | 7 (16.3) | 8 (18.2) |
| 1 or more sleep attacks without sleepiness or drowsiness prior to sleep attack | 6 (14.0) | 11 (25.0) | 2 (4.7) | 6 (13.6) |
| 1 or more sleep attacks with bad outcome | 3 (7.0) | 5 (11.4) | 2 (4.7) | 3 (6.8) |
| Highest number of sleep attacks | | | | |
| 1 | 3 (7.0) | 1 (2.3) | 3 (7.0) | 2 (4.5) |
| 2 | 0 | 4 (9.1) | 0 | 2 (4.5) |
| 3 | 0 | 1 (2.3) | 1 (2.3) | 0 |
| More than 3 | 5 (11.6) | 7 (15.9) | 3 (7.0) | 4 (9.1) |

Abbreviations: LCIG, levodopa-carbidopa intestinal gel; OMT, optimized medical treatment.

**Supplemental Table 2.** Positive screens reported for each MIDI module at baseline and during the 26-week treatment period in the safety population

| **MIDI module, n (%)** | **Baseline** | | **During 26-week treatment** | | |
| --- | --- | --- | --- | --- | --- |
|  | **LCIG**  **n=43** | **OMT**  **n=44** | | **LCIG**  **n=43** | **OMT**  **n=44** |
| Buying disorder screen | 1 (2.3) | 1 (2.3) | | 0 | 2 (4.5) |
| Intermittent explosive disorder | 1 (2.3) | 0 | | 0 | 0 |
| Gambling screen | 1 (2.3) | 1 (2.3) | | 0 | 1 (2.3) |
| Compulsive sexual behavior screen | 1 (2.3) | 0 | | 0 | 2 (4.5) |
| Any MIDI module | 3 (7.0) | 2 (4.5) | | 0 | 4 (9.1) |

Zero positive screens were reported for kleptomania, trichotillomania, and pyromania.

Abbreviations: LCIG, levodopa-carbidopa intestinal gel; OMT, optimized medical treatment; MIDI, Minnesota Impulsive Disorders Interview.

**Supplemental Table 3.** Affirmative responses reported for C-SSRS at baseline and during the 26-week treatment period in the safety population

| **C-SSRS parameter,  n (%)** | **Baseline** | | **During 26-week treatment** | |
| --- | --- | --- | --- | --- |
|  | **LCIG**  **n=43** | **OMT**  **n=44** | **LCIG**  **n=43** | **OMT**  **n=44** |
| **Ideation** | | | | |
| Wish to be dead | 4 (9.3) | 4 (9.1) | 12 (27.9) | 10 (22.7) |
| Nonspecific active suicidal thoughts | 0 | 0 | 5 (11.6) | 3 (6.8) |
| Active thoughts without intent to act | 0 | 0 | 3 (7.0) | 2 (4.5) |
| Patients with suicidal ideation | 4 (9.3) | 4 (9.1) | 12 (27.9) | 10 (22.7) |
| Patients with suicidal ideations only | 4 (9.3) | 4 (9.1) | 11 (25.6) | 10 (22.7) |
| **Behavior** | | | | |
| Actual attempt | 0 | 0 | 1 (2.3) | 0 |
| Preparatory acts or behavior | 0 | 0 | 1 (2.3) | 0 |
| Patients with suicidal behaviors or ideations | 4 (9.3) | 4 (9.1) | 12 (27.9) | 10 (22.7) |

Zero affirmative responses were reported for suicidal ideation categories of active thoughts with some intent (no plan) and active thoughts with plan and intent or for behavior categories of interrupted attempt, aborted attempt, suicidal behavior, completed suicide, patients with suicidal behavior.

Abbreviations: C-SSRS, Columbia-Suicide Severity Rating Scale; LCIG, levodopa-carbidopa intestinal gel; OMT, optimized medical treatment.

**Supplemental Table 4.** Change from baseline to Weeks 12 and 26 in clinical laboratory evaluations and vital signs in the safety population

|  | **Week 12** | | | | | | | **Week 26** | | | | | | |
| --- | --- | --- | --- | --- | --- | --- | --- | --- | --- | --- | --- | --- | --- | --- |
|  | **Change from baseline to Week 12** | | | | **Between-group comparisons  (LCIG vs OMT)** | | | **Change from baseline to Week 26** | | | | **Between-group comparisons  (LCIG vs OMT)** | | |
|  | **LCIG**  **N=42** | | **OMT**  **N=42** | |  |  |  | **LCIG**  **N=42** | | **OMT**  **N=42** | |  |  |  |
| **Variable** | **n** | **Mean (SD)** | **n** | **Mean (SD)** | **LS mean (SE) of difference** | **95% CI** | ***p*-value** | **n** | **Mean (SD)** | **n** | **Mean (SD)** | **LS mean (SE) of difference** | **95% CI** | ***p*-value** |
| Clinical laboratory parameter | | | | | | | | | | | | | | |
| Vitamin B6 (nmol/L) | 40 | –26.8 (68.7) | 41 | –14.2 (89.8) | –12.6  (17.8) | –48.0, 22.9 | 0.483 | 36 | –22.3 (68.5) | 38 | 9.5 (77.0) | –31.8  (17.0) | –65.6, 2.0 | 0.065 |
| Vitamin B12 (pmol/L) | 40 | 29.1 (180.5) | 41 | –19.1 (132.9) | 48.2  (35.2) | –21.8, 118.2 | 0.174 | 37 | 30.8 (236.3) | 38 | –40.8 (174.0) | 71.6  (47.8) | –23.7, 166.9 | 0.139 |
| Serum folate (nmol/L) | 40 | –1.9 (38.5) | 40 | 4.5  (15.7) | –6.3  (6.6) | –19.4, 6.8 | 0.340 | 36 | 2.4 (47.5) | 38 | 2.1 (18.9) | 0.3  (8.3) | –16.3, 16.9 | 0.967 |
| Methylmalonic acid (µg/dL) | 39 | –0.3  (1.9) | 39 | 0.02  (1.7) | –0.3  (0.4) | –1.2, 0.5 | 0.406 | 34 | –0.3 (2.0) | 36 | 0.03 (1.3) | –0.3  (0.4) | –1.1, 0.5 | 0.449 |
| Homocysteine (µmol/L) | 41 | 3.4  (8.4) | 40 | –0.9  (3.9) | 4.3  (1.5) | 1.4, 7.2 | **0.004** | 36 | 4.1 (10.8) | 38 | –1.0 (5.3) | 5.1  (2.0) | 1.2, 9.0 | **0.012** |
| Orthostatic hypotension-related vital signs | | | | | | | | | | | | | | |
| Heart rate (bpm) | 41 | 0.6  (8.1) | 42 | 0.8 (12.0) | –0.2  (2.2) | –4.7, 4.3 | 0.929 | 37 | –0.3 (8.5) | 40 | –1.6 (11.3) | 1.3  (2.3) | –3.3, 5.8 | 0.586 |
| Systolic blood pressure (mmHg) | 41 | 0.1  (13.2) | 42 | 0.5 (13.7) | –0.4  (2.9) | –6.2, 5.5 | 0.904 | 37 | 0.5 (12.2) | 40 | 3.9 (15.1) | –3.4  (3.2) | –9.6, 2.9 | 0.289 |
| Diastolic blood pressure (mmHg) | 41 | –0.8 (10.7) | 42 | 2.9 (13.9) | –3.6  (2.7) | –9.1, 1.8 | 0.186 | 37 | –0.4 (12.0) | 40 | 2.6 (15.1) | -3.0  (3.1) | –9.2, 3.2 | 0.339 |

Significant *p*-values at *p*<0.05 in bold.

Abbreviations: bpm, beats per minute; CI, confidence interval; LCIG, levodopa-carbidopa intestinal gel; LS, least squares; OMT, optimized medical treatment; SD, standard deviation; SE, standard error.

**Supplemental Figure 1.** Change at Week 26 in PGIC in the intent-to-treat population


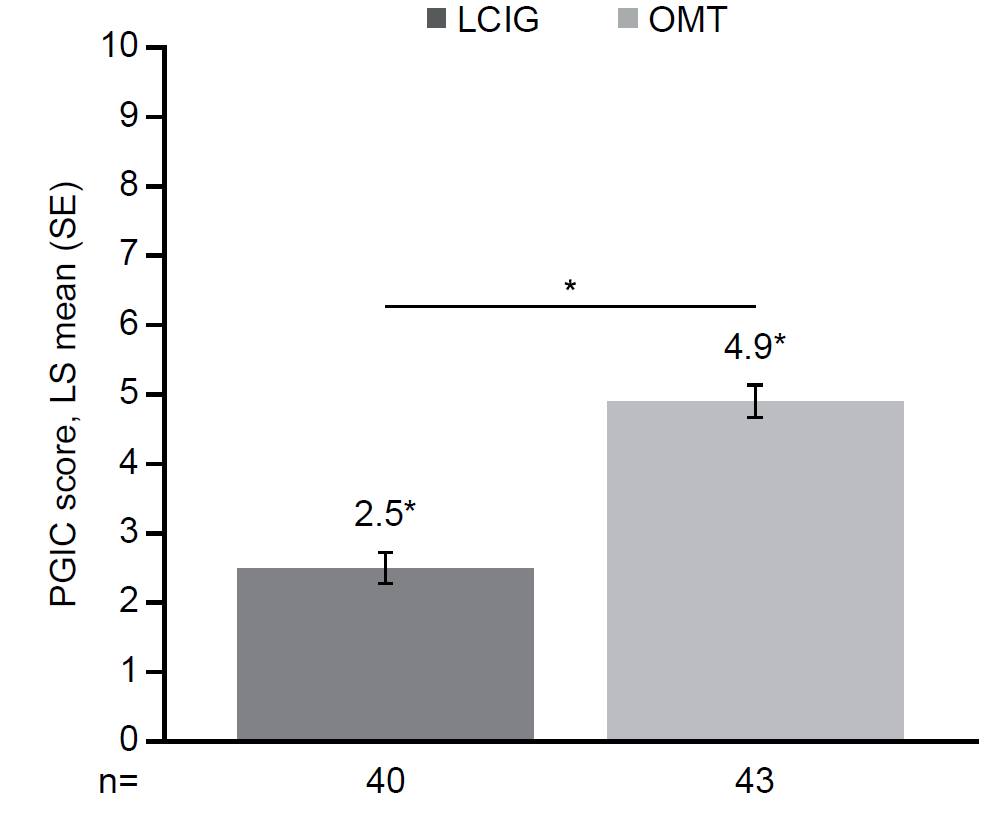


*Significance for between-group differences and within-group differences at Week 26, *p*<0.001.

Abbreviations: LCIG, levodopa-carbidopa intestinal gel; LS, least squares; OMT, optimized medical treatment; PGIC, Patient Global Impression of Change; SE, standard error.

**Supplemental Figure 2.** Change from baseline to Week 26 in KPPS (A) and PAS (B) in the intent-to-treat population

**
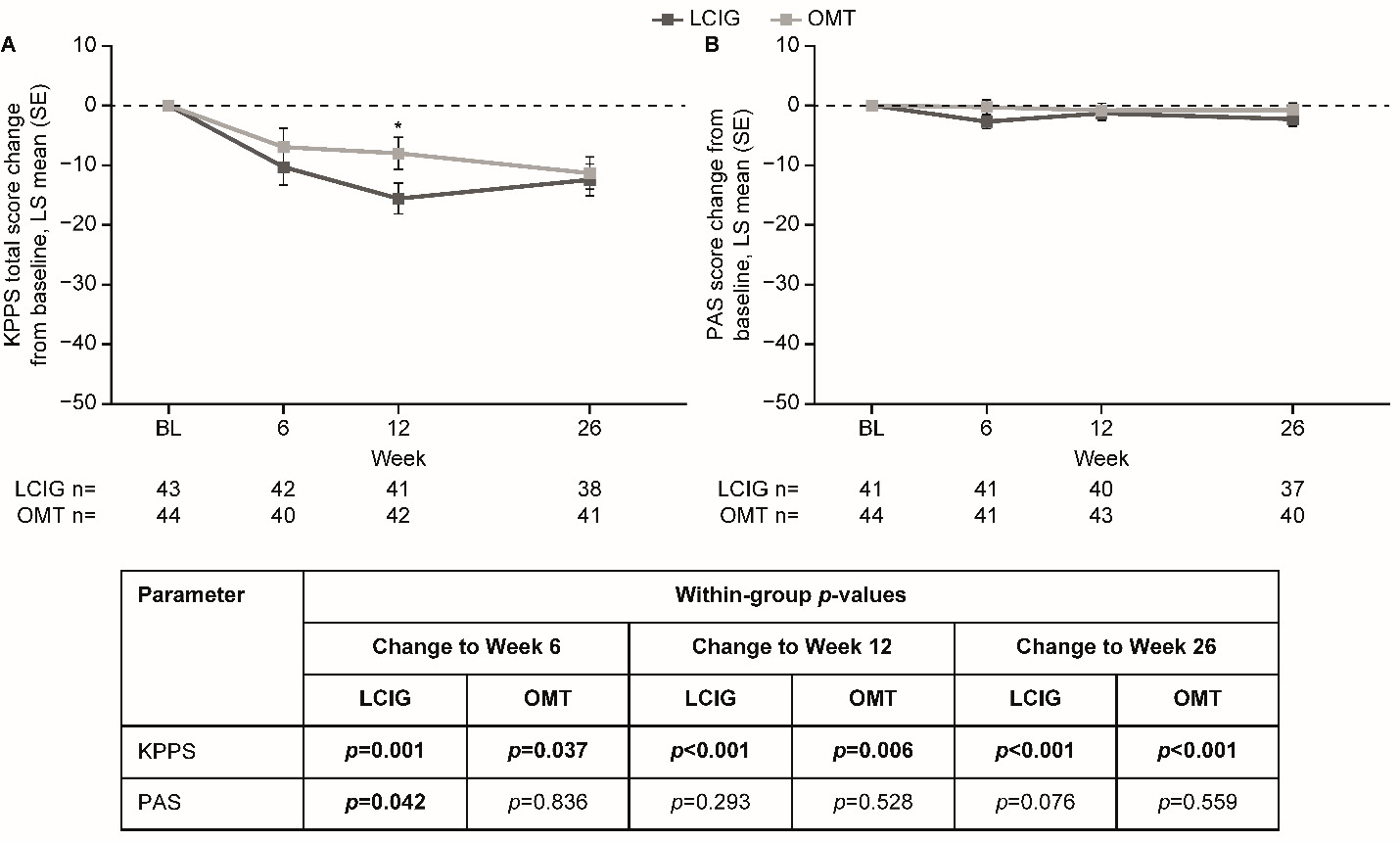
**

*Significance for between-group difference, *p*<0.05.

Within-group differences versus baseline noted in table, with significant *p*-values at *p*<0.05 in bold.

Abbreviations: BL, baseline, KPPS, King’s PD Pain Scale; LCIG, levodopa-carbidopa intestinal gel; LS, least squares; OMT, optimized medical treatment; PAS, Parkinson Anxiety Scale; SE, standard error.
